# SMCKAT, a Sequential Multi-Dimensional CNV Kernel-Based Association Test

**DOI:** 10.3390/life11121302

**Published:** 2021-11-26

**Authors:** Nastaran Maus Esfahani, Daniel Catchpoole, Paul J. Kennedy

**Affiliations:** 1 Australian Artificial Intelligence Institute, University of Technology Sydney, Sydney 2007, Australia; Daniel.Catchpoole@uts.edu.au (D.C.); Paul.Kennedy@uts.edu.au (P.J.K.); 2 The Tumour Bank, The Children’s Hospital at Westmead, Sydney 2145, Australia

**Keywords:** genetic variation, copy number variants, disease-related traits, sequential order, association test

## Abstract

Copy number variants (CNVs) are the most common form of structural genetic variation, reflecting the gain or loss of DNA segments compared with a reference genome. Studies have identified CNV association with different diseases. However, the association between the sequential order of CNVs and disease-related traits has not been studied, to our knowledge, and it is still unclear that CNVs function individually or whether they work in coordination with other CNVs to manifest a disease or trait. Consequently, we propose the first such method to test the association between the sequential order of CNVs and diseases. Our sequential multi-dimensional CNV kernel-based association test (SMCKAT) consists of three parts: (1) a single CNV group kernel measuring the similarity between two groups of CNVs; (2) a whole genome group kernel that aggregates several single group kernels to summarize the similarity between CNV groups in a single chromosome or the whole genome; and (3) an association test between the CNV sequential order and disease-related traits using a random effect model. We evaluate SMCKAT on CNV data sets exhibiting rare or common CNVs, demonstrating that it can detect specific biologically relevant chromosomal regions supported by the biomedical literature. We compare the performance of SMCKAT with MCKAT, a multi-dimensional kernel association test. Based on the results, SMCKAT can detect more specific chromosomal regions compared with MCKAT that not only have CNV characteristics, but the CNV order on them are significantly associated with the disease-related trait.

## 1. Introduction

Genetically speaking, all humans are 99.9 percent the same and the 0.1 percent that makes us all unique is called genetic variation [1]. Genetic variation has two main forms: structural alteration and sequence variation. Copy number variant (CNV) and DNA sequence variation are the most common form of structural alteration and sequence variation in the human genome, respectively [2].

A sequence variation or single nucleotide polymorphism (SNP) represents a difference in a single nucleotide. For example, a SNP may replace the nucleotide cytosine with the nucleotide thymine in a certain stretch of DNA. SNPs are classified into two major types based on the gene region they fall within: coding region and non-coding region. SNPs within a coding sequence do not necessarily change the amino acid sequence of the protein that is produced, due to the degeneracy of the genetic code. SNPs in the coding region are of two types: nonsynonymous and synonymous SNPs. Nonsynonymous SNPs change the amino acid sequence of the protein, while synonymous SNPs do not affect the amino acid sequence of the protein. SNPs do not usually function individually, rather, they work in coordination with other SNPs to manifest a disease or trait [3]. Therefore, many sequence studies have been done to test the association between SNPs and disease or traits.

A copy number variant is the gain or loss of DNA segments in the genome ranging in size from one kilobase to several megabases. CNVs are described by three characteristics: type, chromosomal position, and dosage [4]. The type of CNV is either amplification or deletion. The chromosomal position of the CNV is described by the start and end position of the CNV in the chromosome. The dosage represents the total number of copies of the CNV, with a value less than two relating to deletion and greater than two indicating amplification. Besides, CNVs have phenotypic heterogeneity effects. This means that different CNV types and dosages at the same position in the chromosome can have a different impact. It is reported in biological studies that CNVs are distributed non-randomly in the genomes, in particular they tend to be located close to telomeres and centromeres [5]. However, it is still unclear if there is any specific pattern in the sequential order of CNVs that may lead to a disease or trait.

Association studies have determined that genetic variations, both CNVs and SNPs, are associated with diseases or traits. So, understanding the relationship between genetic variation and disease may provide important insights into genetic causes, leading to effective means in preventing and treating the diseases. While there are lots of computational association studies that have investigated the association between SNPs and diseases or traits, methods for studying CNVs are underdeveloped due to the multi-dimensional characteristics of the CNVs.

The CNV kernel association test (CKAT) [6], copy number profile curve-based association test (CONCUR) [7] and multi-dimensional copy number variant kernel association test (MCKAT) [8] are a few existing computational kernel based methods that have studied the association between CNVs and diseases. In these studies, different kernels are proposed to measure the similarity between CNV profiles with respect to CNV characteristics. Then, the similarity between CNV profiles is compared with those in disease-related trait status to identify any potential association between CNVs and the disease. Among them, our previous method, the MCKAT, is the only method that has incorporated all multidimensional characteristics of CNVs in testing the association between CNVs and disease or trait. The MCKAT calculates the *p*-value of the association test analytically, which is computationally efficient and flexible for CNV association analysis for both rare and common CNV types, as demonstrated in numerical studies. However, neither MCKAT nor other methods consider the CNV sequential order in testing the association between CNVs and disease-related traits.

Starting from MCKAT, we propose a sequential multi-dimensional CNV kernel-based association test (SMCKAT) for investigating the association between CNVs and disease or traits. SMCKAT is not only utilizing all multi-dimensional characteristics of CNVs but also the sequential order of CNVs in testing the association between CNVs and disease or traits. Based on the results, SMCKAT is applicable on both rare and common datasets and capable of identifying hot-spots on the genome where both CNV characteristics and the CNV sequential order are significantly associated with disease or traits.

The rest of this paper is as follows. Section 2 presents the method and materials. Section 3 contains simulation studies. Section 4 shows the results of the real data application. Section 5 presents the discussion, and finally Section 6 concludes the work.

## 2. Method and Materials

We design a sequential multi-dimensional kernel framework capable of measuring the similarity between CNV profiles utilizing all CNV characteristics and the CNV sequential order. It contains two kernels. The first kernel, the pair group kernel, measures the similarity between two groups of CNVs at the same ordinal position of two CNV profiles. It contains three sub-kernels. Each sub-kernel is responsible for measuring the similarity between two CNVs with respect to one of the three CNV characteristics. The second kernel, the whole genome group kernel, aggregates the similarity between every possible CNV pair group to measure the total similarity between the CNV profiles of the subjects. Finally, the association between CNV sequential order across a chromosome and disease-related traits is tested by comparing the similarity in CNV profiles to that in the trait using an association test.

### 2.1. Pair CNV Group Kernel

**Figure 1:**
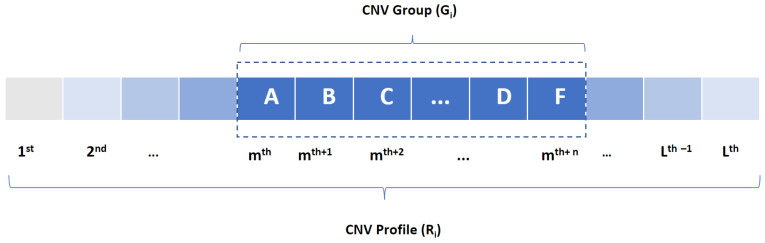
Generating CNV profile $R_{i}$ where CNVs are sorted with respect to their chromosomal position. A, B,…, and F are arbitrary CNVs at $m^{th}$, $m^{th+1}$, …, and $m^{th+n}$ positions and $G_{i}$ is a group of CNVs of size *n*.

Let *X* denote a single CNV which is defined by four characteristics as $X=(X^{\left(1\right)},X^{\left(2\right)}$, $X^{\left(3\right)},X^{\left(4\right)})$ where $X^{\left(1\right)}$ and $X^{\left(2\right)}$ are the CNV starting and ending position on the chromosome, $X^{\left(3\right)}$ is the CNV type, and $X^{\left(4\right)}$ is the CNV dosage. First, we generate the CNV profile *R* for subject *i* with *l* CNVs as $R_{i}=\left(X_{1}^{i},X_{2}^{i},\ldots ,X_{l_{i}}^{i}\right)$ where CNVs are sorted based on their chromosomal position. Secondly, we extract a CNV group of size *n* out of the CNV profile as $G_{i}=\left(X_{m}^{i},X_{m+1}^{i},\ldots ,X_{m+n}^{i}\right)$ where *n* is the group size that can take any value between 1 and *l*, the number of existing CNVs in a CNV profile as is shown in Figure 1.

**Figure 2:**
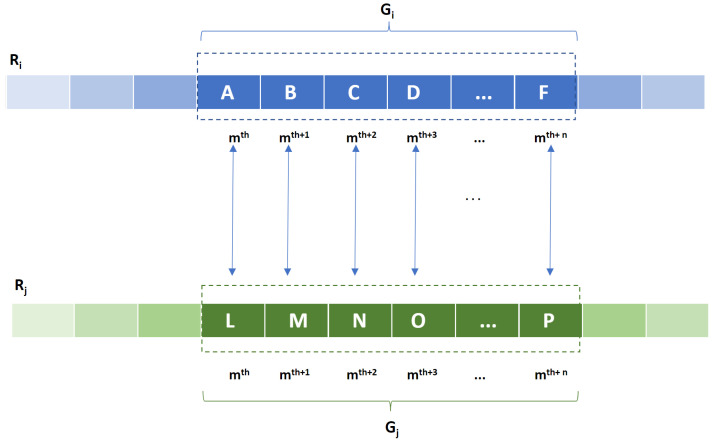
Aligning CNVs within two CNV groups of size *n*, $G_{i}$ and $G_{j}$, to generate *n* CNV pairs.

We propose a pair CNV group kernel, $K_{PG}$, to measure the similarity between two CNV groups of size *n*, $G_{i}$ and $G_{j}$, in two CNV profiles. First, $K_{PG}$ aligns each CNV in the $G_{i}$ with its relevant CNV in the $G_{j}$ with respect to their position to generate *n* CNV pairs as is shown in Figure 2.

Then, $K_{PG}$ measures the similarity between each CNV pair using the single pair CNV kernel, $K_{S}$, we proposed in [8]. $K_{S}$ measures the similarity between a CNV pair by three sub-kernels considering all CNV features including chromosomal position, type and dosage. Finally, $K_{PG}$ averages the similarities calculated by $K_{S}$ between all generated CNV pairs to measure the similarity between two CNV groups, $G_{i}$ and $G_{j}$, as

(1) $$K_{PG}\left(G_{i},G_{j}\right)=\sum_{m=1}^{n}\frac{K_{s}\left(X_{m}^{i},X_{m}^{j}\right)}{n}$$

where $K_{s}$ is defined as

(2) $$\begin{array}{c} K_{s}\left(X_{m}^{i},X_{m}^{j}\right)=\left[\frac{Intersection\left(\left(X_{m}^{i\left(1\right)},X_{m}^{i\left(2\right)}\right),\left(X_{m}^{j\left(1\right)},X_{m}^{j\left(2\right)}\right)\right)}{Union\left(\left(X_{m}^{i\left(1\right)},X_{m}^{i\left(2\right)}\right),\left(X_{m}^{j\left(1\right)},X_{m}^{j\left(2\right)}\right)\right)}\right]\times \\ \left[\frac{\left(X_{m}^{i\left(3\right)}==X_{m}^{j\left(3\right)}\right)+1}{2}\right]\times \left[\frac{1}{2^{\left|DR\left(X_{m}^{i\left(4\right)}\right)-DR\left(X_{m}^{j\left(4\right)}\right)\right|}}\right] \end{array}$$

and the first term measures the mutual presence of a CNV with a specific start and end position by dividing the size of the intersection of two CNVs to their union size. The intersection function calculates the length of the chromosomal region that belongs to both CNVs. Similarly, the union function calculates the length of the chromosomal region that consists of both regions that belong to the first CNV and to the second CNV. The second term compares the CNV type of two CNVs to calculate the similarity between them. The third term measures the similarity between two CNVs with respect to their dosage. The DR is the difference from the reference function we proposed in [8] as DR(dosage)=|dosage−2|. DR measures the difference between a CNV dosage and the reference dosage value 2.

### 2.2. Whole Genome CNV Group Kernel

**Figure 3:**
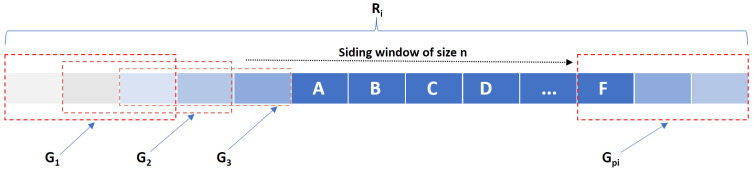
Sliding window of size *n* across CNV profile to extract CNV groups of size *n*.

First, we create a window of size *n*. We slide this window across the CNV profile $R_{i}$ as is shown in Figure 3 to extract all possible CNV groups of size *n* as $P_{i}=\left(G_{1}^{i},\ldots ,G_{p_{i}}^{i}\right)$ where CNV groups are sorted based on their position and $p_{i}$ is the number of extracted CNV groups for the CNV profile $R_{i}$. Similarly, we have another CNV group series $P_{j}=\left(G_{1}^{j},\ldots ,G_{q_{j}}^{j}\right)$ for CNV profile $R_{j}$.

**Figure 4:**
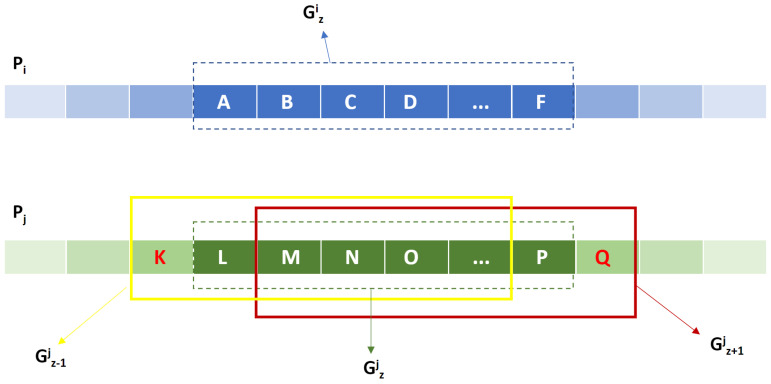
Aligning $G_{z}^{i}$ to the best group-to-group correspondence of the highest similarity among $G_{z-1}^{j}$, $G_{z}^{j}$ and $G_{z+1}^{j}$.

Then, we propose the whole genome CNV group kernel, $K_{WG}$, to measure the similarity between two CNV group series $P_{i}$ and $P_{j}$ as

(3) $$\begin{array}{c} K_{WG}\left(P_{i},P_{j}\right)=\left\{\begin{array}{c} 0\;\;\;\;\;\;\;\;\;\;\;\;\;\;\;\;\;\;\;\;\;\;\;\;\;\;\;\;\;\;\;\;\;\;\;\;\;\;\;\;\;\;\;\;\;\;\;\;\;\;\;\;\;\;\mathrm{if}\;p_{i}\times q_{i}=0\\ \sum_{z=1}^{Max(p_{i},q_{i})}Max(K_{PG}\left(G_{z}^{i},G_{z-1}^{j}\right),\\ K_{PG}\left(G_{z}^{i},G_{z}^{j}\right),K_{PG}\left(G_{z}^{i},G_{z+1}^{j}\right))\;\;\;\;\;\;\;\;\;\;\mathrm{if}\;p_{i}\times q_{i}\neq 0 \end{array}\right. \end{array}$$

where $K_{PG}\left(.,.\right)$ is the pair CNV group kernel from (1). $K_{WG}$ measures the similarity between the pair CNV groups of the same position and aggregates these similarities to calculate the similarity in two CNV group series. The second maximum operation in the definition of $K_{WG}$ searches for the best group-to-group correspondence of the highest similarity to align CNV groups in two CNV group series as is shown in Figure 4.

The kernel-based association test described in the following section, requires a kernel similarity matrix *K*. *K* is a d×d matrix, where $K_{ij}=K_{WG}\left(P_{i},P_{j}\right)$ and *d* is the number of existing CNV profiles. $K_{ij}$ expresses the similarity between CNV profile *i* and *j* measured by $K_{WG}$.

### 2.3. Kernel-Based Association Test

We use the following logistic regression model to test the association between CNV sequential order and a disease related trait

(4) $$\begin{array}{c} logit\left[Pr\left(y_{i}=1\right)\right]=\beta_{0}+Z\beta +f\left(P_{i}\right) \end{array}$$

where $y_{i}$ is the status of the disease related trait with $y_{i}=1$ denoting the existence of the trait and $y_{i}=0$ denoting otherwise, and i=1,2,…,d indexing the CNV profiles, and *Z* is the covariate matrix including information such as age and gender. $P_{i}$ is the CNV group series of the profile $R_{i}$ as explained previously. f(·) is a function spanned by the whole genome CNV group kernel $K_{WG}\left(\cdot ,\cdot \right)$. According to Equation (4), the hypothesis of no association between the CNV sequential order and the existence of a disease related trait can be tested as $H_{0}:f\left(\cdot \right)=0$. To test this, one way is to treat the f(·) as a random effect vector which is distributed as N(0,τK), where τ≥0 and *K* is the d×d similarity matrix, treated as covariance matrix of the random effect, generated by $K_{WG}$ as defined in [6]. Liu et al. [9] has shown that testing $H_{0}:f\left(\cdot \right)$ is equivalent to testing $H_{0}:\tau =0$ in the logistic mixed effect model. Moreover, τ is a variance component parameter in the logistic mixed effect model, which can be tested using a restricted maximum likelihood-based score test [9,10].

We use the following score test statistic where $\hat{y}$ is estimated under the null model $logit\left[Pr\left(y_{i}=1\right)\right]=\beta_{0}+Z\beta$ and *K* is the similarity matrix explained in the previous section.

(5) $$Q={\left(y-\;\hat{y}\right)}^{\prime }K\left(y-\;\hat{y}\right)$$

**Figure 5:**
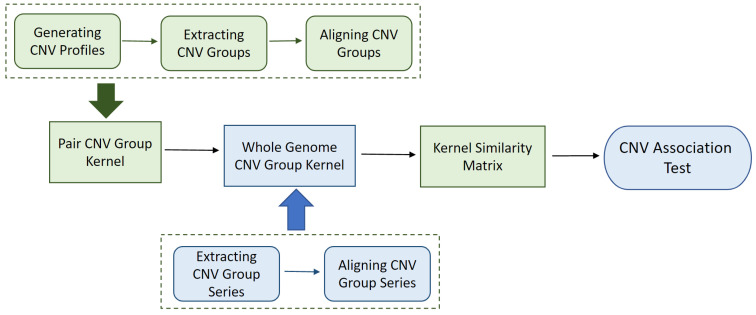
SMCKAT workflow diagram.

Then, we used the Davies method [11] as implemented in the CKAT R package [6] to calculate the *p*-value of the proposed kernel based association test. The SMCKAT workflow is summarized in Figure 5

### 2.4. Common and Rare CNV Data

Biologists generally assign CNVs to one of two major types, depending on the length of the affected chromosomal region and occurrence frequency: copy number polymorphisms (CNPs) and rare variants [4]. CNPs are widespread in the general population, with an average occurrence frequency greater than one percent while rare variants are much longer than CNPs, ranging from hundreds of thousands of base pairs to over one million base pairs.

We apply SMCKAT on both rare and common CNV public domain genome sequencing data sets to evaluate the performance on both CNV types. The two CNV data sets used in this study are from individuals with rhabdomyosarcoma (RMS) cancer and autism spectrum disorder (ASD). The RMS data set [12] contains the common CNVs for 44 subjects, while the ASD data set [13] has the rare CNVs of 588 subjects. In both data sets, each CNV is presented by chromosomal position, type, and dosage.

## 3. Simulation Studies

We conducted simulations to evaluate the performance of SMCKAT and ensure that it can properly handle type I and II errors, as well as having relatively high power in detecting existing associations. Besides SMCKAT, the MCKAT and CKAT are also studied. We conduct our simulation studies under two main scenarios. In the first scenario, we evaluate the performance of the SMCAKT on the rare CNV data. In the second scenario, we evaluate the performance of the SMCKAT on the common CNV data.

We use the ASD dataset and the RMS dataset in the first and second simulation scenarios, respectively. These datasets are studied in the real data analysis and further details regarding them are shared in the Section 4. We simulated $10^{5}$ datasets for each simulation scenario.

The ASD dataset has the same dosage value for all deletions and similarly the same dosage value for all amplifications. Therefore, we randomly generate other values for the CNV dosage to conduct our simulation studies and investigate the dosage effect in identifying existing associations. The simulated dosage value can take 0 or 1 for deletion types and 3, 4, …, 7 for amplification types. We use equal probabilities when generating random dosage values for deletion and amplification, 0.5 and 0.2, respectively.

A case-control phenotype is generated for both SMCKAT and MCKAT from the following logistic model that we proposed in [8],

(6) $$\begin{array}{c} logit\left(Pr\left(Y_{i}=1\right)\right)=\beta_{0}+\sum_{j=1}^{m_{i}}\beta_{j}^{Len}\left(X_{ij}^{\left(2\right)}-X_{ij}^{\left(1\right)}\right)+\sum_{j=1}^{m_{i}}\left(\beta_{j}^{Del}I\left[X_{ij}^{\left(3\right)}=1\right]+\beta_{j}^{Amp}I\left[X_{ij}^{\left(3\right)}=3\right]\right)\\ +\sum_{j=1}^{m_{i}}\beta_{j}^{Dsg}\left|X_{ij}^{\left(4\right)}-2\right|+\sum_{j=1}^{m_{i}}\beta_{j}^{Len*Del*Dsg}\left(X_{ij}^{\left(2\right)}-X_{ij}^{\left(1\right)}\right)\times I\left[X_{ij}^{\left(3\right)}=1\right]\times X_{ij}^{\left(4\right)}\\ +\sum_{j=1}^{m_{i}}\beta_{j}^{Len*Amp*Dsg}\left(X_{ij}^{\left(2\right)}-X_{ij}^{\left(1\right)}\right)\times I\left[X_{ij}^{\left(3\right)}=3\right]\times X_{ij}^{\left(4\right)} \end{array}$$

where $X_{ij}=\left(X_{ij}^{\left(1\right)},X_{ij}^{\left(2\right)},X_{ij}^{\left(3\right)},X_{ij}^{\left(4\right)}\right)$ is the *j*th CNV of the *i*th individual as defined previously. $\beta_{0}$ corresponds to a baseline disease rate. $\beta_{j}^{Len}$ controls the effect of chromosomal position, and $\beta_{j}^{Del}$ and $\beta_{j}^{Dup}$ are the log ratio of a CNV *j* for being deletion versus amplification and vice versa. $\beta_{j}^{Del}$ and $\beta_{j}^{Dup}$ share the same values but different signs. $\beta_{j}^{Len*Amp*Dsg}$ and $\beta_{j}^{Len*Del*Dsg}$ allow the effect of the chromosomal position and CNV type to differ by dosage in CNV *j*.

After generating phenotypes for SMCKAT and MCKAT, we use following logistic model that is proposed in [6] to generate the phenotypes under CKAT method:

(7) $$logit\left(\pi_{i}\right)=\beta_{0}+\sum_{j=1}^{m_{i}}\left(\beta_{j}^{Del}I\left[X_{ij}^{\left(2\right)}=1\right]+\beta_{j}^{Dup}I\left[X_{ij}^{\left(2\right)}=3\right]\right)X_{ij}^{\left(1\right)}$$

where $X_{ij}=\left(X_{ij}^{\left(1\right)},X_{ij}^{\left(2\right)}\right)$ is the *j*th CNV of *i*th subject, $\pi_{i}=Pr\left(Y_{i}=1\right)$, $\beta_{0}$ is the prevalence rate of the disease, and $\beta_{j}^{Dup}$, $\beta_{j}^{Del}$ are the log of the odd ratio of CNV *j* for duplication and deletion respectively.

### Simulation Results

**Figure 6:**
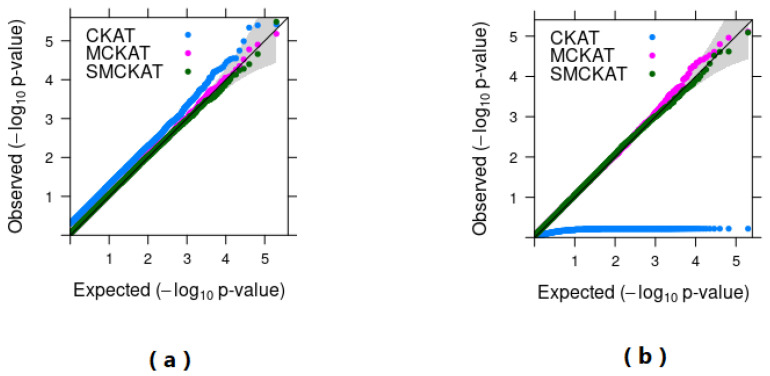
*p*-value based QQ-plots of MCKAT and CKAT under first (**a**) and second (**b**) simulation scenario.

The QQ-plots of *p*-values of SMCKAT, MCKAT and CKAT under both simulation scenarios are presented in Figure 6.

Based on the QQ-plot (a), SMCKAT and MCKAT are on the 45 degree line under the first simulation scenario. This indicates that both SMCKAT and MCKAT can properly handle the type I and II error rate under different nominal significance levels even as low as $10^{-5}$ when dealing with the rare CNV dataset. However, CKAT is showing a higher chance of committing the type II error in detecting existing associations between the rare CNVs and phenotype.

As is shown in QQ-plot (b), both SMCKAT and MCKAT can protect the correct type I and II error rate at different nominal significance levels in dealing with the common CNV data. We observe that SMCKAT is a little conservative when the significance level is small. However, CKAT shows a weak performance in handling the type I error and detecting existing associations between the common CNVs and phenotype.

**Figure 7:**
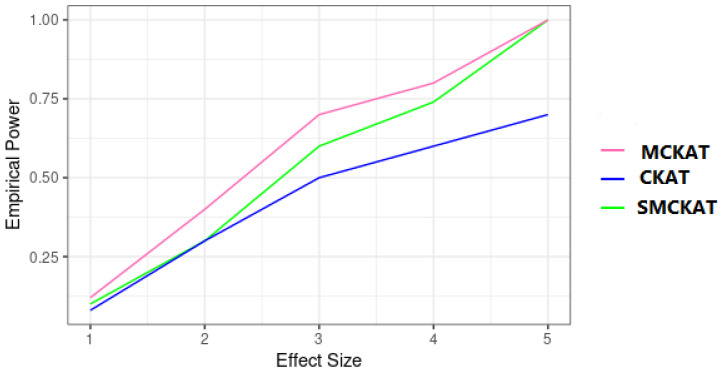
Empirical power of SMCKAT, MCKAT and CKAT under first simulation scenario, rare CNV data.

**Figure 8:**
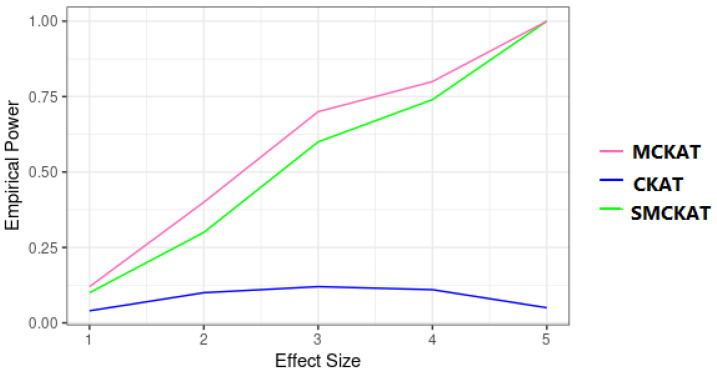
Empirical power of SMCKAT, MCKAT and CKAT under second simulation scenario, common CNV data.

The empirical powers of SMCKAT, MCKAT and CKAT under the first and second scenarios are presented in Figure 7 and Figure 8, respectively. As is shown is Figure 7, SMCKAT and MCKAT have almost similar powers when dealing with rare CNVs. However, CKAT shows lower power compared with SMCKAT and MCKAT. The reason might be that the CKAT is not considering the CNV dosage information when testing the association.

Similarly, in the second simulation scenario, SMCKAT and MCKAT have similar powers. However, CKAT is showing low power when dealing with common CNV data. This might be due to the CKAT scanning algorithm for aligning CNVs in the CNV profiles. The CKAT shift-by-one scanning algorithm can capture similarity between limited number of CNVs, which may result in low performance when dealing with common CNVs.

## 4. Real Data Application Results

We conducted SMCKAT analysis, for different CNV group sizes, on single chromosomes and the whole genome to test the association between CNV sequential order and disease-related traits. The disease-related traits studied in this paper are cancer subtype for the RMS data set and disease status for the ASD data set. We compared SMCKAT results with those obtained from MCKAT and CKAT to evaluate SMCKAT performance on real CNV data.

### 4.1. CNV Analysis on Rhabdomyosarcoma Data Set

First, we conducted the experiment on the RMS data. The RMS occurs as two major histological subtypes, embryonal (ERMS) and alveolar (ARMS). The classification of the RMS subtype has a direct effect on the patient treatment options. The RMS data includes a total of 59,131 CNVs for 25 alveolar and 19 embryonal cancers. We apply SMCKAT to each of 23 chromosome pairs, with different CNV group sizes, to test the association between CNV sequential order and RMS subtype. Bonferroni correction is used for adjusting the multiple testing to control the family-wise error rate (FWER) of α=0.05. Since 22 chromosomes and a sex chromosome are being tested, the *p*-value threshold for a whole-chromosome significance is calculated as $0.05/23=2.2\times 10^{-3}$. SMCKAT identifies four chromosomes out of the existing 23 chromosomes that have a CNV sequential order that is significantly associated with the RMS sub-type. The *p*-values of SMCKAT for these four chromosomes are reported in Table 1.

Based on the results, SMCKAT identifies CNV sequential order in chromosomes 2, 8, 11, and 13, significantly associated with distinguishing RMS subtype at FWER= $2.2\times 10^{-3}$. These results are consistent with the existing biological knowledge, which shows the ability of the SMCKAT to identify the CNV sequential order significantly associated with specific disease-related traits.

For example, ref. [14] shows that RMS is associated with specific chromosomal abnormalities that differentiate ARMS and ERMS. Based on their study, approximately 80% of ARMS tumors display a translocation between the FOXO1 transcription factor gene located on chromosome 13 and the PAX3 transcription factor gene on chromosome 2, and ERMS tumors show a higher frequency of specific genetic mutation on chromosome 11 than ARMS. Ref. [15] has revealed the same earlier. Furthermore, ref. [16] has found that the ARMS subtype is significantly associated with amplifications on chromosome 8. Our findings show another mechanism like CNVs can play a significant role in causing any disease-related traits besides gene mutations and chromosomal translocations.

We tested different CNV group sizes when applying SMCKAT to the RMS data set. Based on the results reported in Table 1, SMCKAT shows the strongest evidence and smallest *p*-value for the chromosome 8 for all CNV group sizes. It means subjects with the same RMS subtype may have a similar CNV sequential order on their chromosome 8.

We tested SMCKAT on the RMS data set for group sizes greater than six. We observed an increasing trend in *p*-values by increasing the group size, which shows a decline in the significance level of the CNV sequential order associated with the RMS subtype.

### 4.2. CNV Analysis on Cytogenetic Bands in RMS

Based on the result reported in Table 1, there is strong statistical evidence, as supported by a *p*-value near to zero, that the CNV sequential order of chromosome 8 with group size of six is significantly associated with the RMS subtype. Therefore, we picked chromosome 8 with a CNV group size of six for further analysis. We partitioned chromosome 8 into smaller regions based on the cytogenetic bands. We applied SMCKAT on each cytogenetic band to check if SMCKAT is capable of detecting more specific regions rather than chromosomes. Then, we compared the results with of MCKAT and CKAT. Table 2 contains the *p*-values of the association test in each cytogenetic band in chromosome 8. Since 40 cytogenetic bands are being tested in chromosome 8, the *p*-value threshold for a band significance is calculated as $0.05/40=1.2\times 10^{-3}$.

As shown in Table 2, both SMCKAT and MCKAT detect significantly associated cytogenetic bands with the RMS subtype while CKAT does not identify any significant regions. MCKAT has identified 8 cytogenetic bands that CNVs on them are significantly associated with RMS sub type. Two out of these eight cytogenetic bands, 8p23.1 and 8p12.0, are identified by SMCKAT as well. It means not only the CNV characteristics but also the CNV sequential order in these two bands are significantly associated with the RMS sub type. Based on the results, SMCKAT has the potential to provide us with more specific CNV regions when we are testing the association between CNVs and disease-related traits compared with MCKAT.

### 4.3. CNV Analysis on Autism Data Set

We applied SMCKAT on the ASD data set to evaluate its performance on the rare CNV type. We aimed to test if there was any association between the sequential order of CNVs and ASD status. The ASD data set contains 1285 rare CNVs on 310 individuals with ASD and 1074 rare CNVs on 278 healthy individuals. Since the ASD data set contains only rare and large CNVs, an arbitrary CNV profile may have no or few CNVs on some chromosomes. Therefore, instead of applying SMCKAT to every 23 chromosomes, we applied it to the whole genome. Then, we tested if there is any association between the whole genome CNV sequential order and the ASD status. We considered 0.05 as the *p*-value threshold for the whole-chromosome significance. As is reported in Table 3, there is strong statistical evidence, up to a CNV group size of five, that subjects with the same disease status have similar CNV ordesr in their CNV profiles. We tested SMCKAT on the ASD data for the larger group sizes as well. We observed an increasing trend in *p*-values by increasing the group size, which shows a decline in the significance level of the CNV sequential order associated with the ASD status.

## 5. Discussion

SMCKAT tests the association between CNVs and disease-related traits. It checks if CNVs are randomly distributed on the chromosomes or if their sequential orders are significant and have associations with disease-related traits. Our approach has several advantages over the existing methods. First, it measures the similarity between CNV profiles by considering not only all CNV characteristics but also the CNV sequential order. To our knowledge, it is the first approach to study the association between CNV sequential order and disease related traits. Secondly, it is applicable to both rare and common CNV data sets, while previous methods like CKAT can not deal with common CNV data sets. Thirdly, SMCKAT is more stringent when compared with the state-of-the-art approach MCKAT in detecting significant CNV regions. Finally, SMCKAT can help biologists detect significantly associated CNV regions with any disease-related trait across a patient group instead of examining the CNVs case by case in each subject.

Although our experimental results are promising and more specific compared with the state-of-the-art kernel approach, this study has limitations. There are not many publicly available CNV data sets. Besides, most available ones do not contain all CNV features together, in particular the dosage information. Consequently, our method is tested only on one data set, an RMS data set, that includes all multi-dimensional CNV characteristics. For the ASD data set, we considered a dosage less than two for all deletions and greater than two for all amplifications to make the most of the proposed method’s capability. Applying SMCKAT to more data sets containing all CNV characteristics can help to determine its strengths and weaknesses. In addition, there is no existing study, neither biological nor computational, that has studied the CNV sequential order to be able to validate our experimental results with it.

Our study shows that CNV sequential order has the potential to play a significant role in causing disease-related traits, but more new findings can be revealed by conducting more comprehensive analysis upon the availability of data.

## 6. Conclusions

This paper presents a sequential multi-dimensional CNV association test identifying associations between CNVs and disease-rated traits using all multi-dimensional CNV characteristics and CNV sequential order. Our method, SMCKAT, uses different kernels to measure the similarity between CNV profiles with respect to both CNV orders and characteristics. Then, the similarity in CNV profiles is compared to the similarity in disease-related traits to test for an association.

The evaluation was conducted on two types of CNV data sets, a rare CNV data set and a common CNV data set. Results indicate that our method provides statistically strong evidence that there is an association between the sequential order of CNVs and disease related traits. Currently, SMCKAT is capable of testing the association between CNVs and qualitative disease-rated traits. In our future work, we will expand the SMCKAT framework to be applicable to both qualitative and quantitative traits.

## Figures and Tables

**Table 1:** *p*-values of testing the association between CNV sequential order and RMS subtype trying different CNV group sizes. *n* is the group size and (#) denotes the total number of CNVs on the chromosome.

Chr.	#CNV	*n* = 1	*n* = 2	*n* = 3	*n* = 4	*n* = 5	*n* = 6
2	5584	$2.45\times 10^{-2}$	$5.10\times 10^{-2}$	$8.31\times 10^{-2}$	$3.49\times 10^{-3}$	$4.25\times 10^{-3}$	$3.21\times 10^{-2}$
8	5365	$2.61\times 10^{-5}$	$7.37\times 10^{-6}$	$1.13\times 10^{-6}$	$7.63\times 10^{-7}$	$4.99\times 10^{-8}$	0
11	3449	$2.03\times 10^{-2}$	$8.26\times 10^{-3}$	$2.93\times 10^{-3}$	$1.54\times 10^{-3}$	$5.82\times 10^{-4}$	$1.20\times 10^{-4}$
13	2462	$1.80\times 10^{-3}$	$3.56\times 10^{-3}$	$4.86\times 10^{-3}$	$6.06\times 10^{-3}$	$7.89\times 10^{-3}$	$6.23\times 10^{-2}$

**Table 2:** *p*-values of the testing association between RMS subtype and CNVs in the chromosome 8 cytogenetic bands by SMCKAT, MCKAT and CKAT. (*) denotes significant association between RMS subtype and CNVs, (#) denotes the number of total CNVs on the band.

Arm	Band		Start	Stop	#CNVs	SMCKAT		MCKAT		CKAT	
p	23	3	1	2,300,000	113	9	$6\times 10^{-2}$	3	$4\times 10^{-4}$ *	4	$917\times 10^{-1}$
p	23	2	2,300,001	6,300,000	85	3	$0\times 10^{-2}$	2	$0\times 10^{-2}$	3	$939\times 10^{-1}$
p	23	1	6,300,001	12,800,000	304	1	$8\times 10^{-4}$ *	4	$7\times 10^{-8}$ *	4	$755\times 10^{-1}$
p	22	0	12,800,001	19,200,000	101	2	$8\times 10^{-2}$	8	$2\times 10^{-3}$	4	$327\times 10^{-1}$
p	21	3	19,200,001	23,500,000	102	1	$1\times 10^{-1}$	2	$5\times 10^{-2}$	4	$237\times 10^{-1}$
p	21	2	23,500,001	27,500,000	82	3	$4\times 10^{-2}$	3	$6\times 10^{-2}$	4	$717\times 10^{-1}$
p	21	1	27,500,001	29,000,000	50	2	$5\times 10^{-2}$	1	$6\times 10^{-2}$	4	$948\times 10^{-1}$
p	12	0	29,000,001	36,700,000	190	1	$3\times 10^{-6}$ *	3	$7\times 10^{-5}$ *	4	$658\times 10^{-1}$
p	11	23	36,700,001	38,500,000	48	1	0	3	$7\times 10^{-3}$	3	$916\times 10^{-1}$
p	11	22	38,500,001	39,900,000	57	9	$3\times 10^{-2}$	8	$4\times 10^{-3}$	4	$613\times 10^{-1}$
p	11	21	39,900,001	43,200,000	147	4	$4\times 10^{-3}$	1	$0\times 10^{-4}$ *	3	$655\times 10^{-1}$
p	11	1	43,200,001	45,200,000	72	8	$8\times 10^{-2}$	2	$8\times 10^{-2}$	4	$584\times 10^{-1}$
q	11	1	45,200,001	47,200,000	41	1	0	2	$1\times 10^{-2}$	4	$436\times 10^{-1}$
q	11	21	47,200,001	51,300,000	200	4	$4\times 10^{-3}$	8	$4\times 10^{-5}$ *	4	$064\times 10^{-1}$
q	11	22	51,300,001	51,700,000	6	9	$3\times 10^{-1}$	4	$7\times 10^{-2}$	4	$200\times 10^{-1}$
q	11	23	51,700,001	54,600,000	61	1	0	6	$1\times 10^{-2}$	4	$657\times 10^{-1}$
q	12	1	54,600,001	60,600,000	177	9	$1\times 10^{-3}$	7	$0\times 10^{-4}$ *	4	$505\times 10^{-1}$
q	12	2	60,600,001	61,300,000	18	1	0	3	$3\times 10^{-2}$	4	$502\times 10^{-1}$
q	12	3	61,300,001	65,100,000	134	4	$9\times 10^{-2}$	1	$1\times 10^{-2}$	4	$110\times 10^{-1}$
q	13	1	65,100,001	67,100,000	71	4	$4\times 10^{-2}$	5	$8\times 10^{-3}$	4	$427\times 10^{-1}$
q	13	2	67,100,001	69,600,000	54	5	$8\times 10^{-2}$	4	$3\times 10^{-3}$	4	$659\times 10^{-1}$
q	13	3	69,600,001	72,000,000	62	1	$4\times 10^{-2}$	1	$8\times 10^{-3}$	3	$762\times 10^{-1}$
q	21	11	72,000,001	74,600,000	144	4	$8\times 10^{-1}$	8	$4\times 10^{-3}$	3	$325\times 10^{-1}$
q	21	12	74,600,001	74,700,000	1	1	0	1	0	1	0
q	21	13	74,700,001	83,500,000	308	1	$0\times 10^{-2}$	2	$6\times 10^{-3}$	4	$927\times 10^{-1}$
q	21	2	83,500,001	85,900,000	56	4	$8\times 10^{-2}$	2	$9\times 10^{-2}$	4	$189\times 10^{-1}$
q	21	3	85,900,001	92,300,000	185	4	$7\times 10^{-3}$	1	$0\times 10^{-4}$ *	4	$215\times 10^{-1}$
q	22	1	92,300,001	97,900,000	182	1	$7\times 10^{-2}$	1	$0\times 10^{-2}$	3	$072\times 10^{-1}$
q	22	2	97,900,001	100,500,000	103	4	$5\times 10^{-2}$	3	$9\times 10^{-3}$	4	$395\times 10^{-1}$
q	22	3	100,500,001	105,100,000	162	1	$2\times 10^{-2}$	4	$6\times 10^{-3}$	4	$458\times 10^{-1}$
q	23	1	105,100,001	109,500,000	135	2	$8\times 10^{-3}$	2	$5\times 10^{-3}$	4	$017\times 10^{-1}$
q	23	2	109,500,001	111,100,000	33	9	$8\times 10^{-1}$	8	$0\times 10^{-1}$	3	$005\times 10^{-1}$
q	23	3	111,100,001	116,700,000	185	1	$1\times 10^{-2}$	2	$3\times 10^{-3}$	4	$419\times 10^{-1}$
q	24	11	116,700,001	118,300,000	53	4	$6\times 10^{-2}$	2	$6\times 10^{-2}$	4	$705\times 10^{-1}$
q	24	12	118,300,001	121,500,000	109	2	$5\times 10^{-3}$	2	$2\times 10^{-3}$	4	$068\times 10^{-1}$
q	24	13	121,500,001	126,300,000	151	2	$2\times 10^{-2}$	6	$0\times 10^{-3}$	4	$856\times 10^{-1}$
q	24	21	126,300,001	130,400,000	208	5	$0\times 10^{-2}$	1	$9\times 10^{-2}$	3	$922\times 10^{-1}$
q	24	22	130,400,001	135,400,000	155	5	$5\times 10^{-2}$	1	$5\times 10^{-2}$	4	$638\times 10^{-1}$
q	24	23	135,400,001	138,900,000	162	2	$8\times 10^{-1}$	7	$7\times 10^{-3}$	4	$512\times 10^{-1}$
q	24	3	138,900,001	145,138,636	354	8	$8\times 10^{-3}$	2	$5\times 10^{-8}$ *	4	$277\times 10^{-1}$

**Table 3:** *p*-values of testing the association between CNV sequential order and ASD status trying different CNV group sizes.

*n*	1	2	3	4	5	6
*p*-value	0	$7.91\times 10^{-9}$	$3.09\times 10^{-6}$	$3.62\times 10^{-4}$	$4.89\times 10^{-3}$	$1.03\times 10^{-1}$

## Data Availability

The ASD and RMS datasets supporting the conclusions of this article are available at https://www.ncbi.nlm.nih.gov/pmc/articles/PMC3213131 and https://www.ncbi.nlm.nih.gov/gap (accession number: phs000720.v3.p1) respectively (access date: 20 November 2021). The SMCKAT R package is publicly available at https://github.com/nesfehani/SMCKAT GitHub repository (access date: 20 November 2021).

## References

[1] National Human Genome Research Institute (2018). Genetics vs. Genomics Fact Sheet.

[2] Frazer K.A., Murray S.S., Schork N.J., Topol E.J. (2009). Human genetic variation and its contribution to complex traits. Nat. Rev. Genet..

[3] Edwards D., Forster J.W., Chagné D., Batley J. (2007). What Are SNPs?. Association Mapping in Plants.

[4] Schrider D.R., Hahn M.W. (2010). Gene copy-number polymorphism in nature. Proc. R. Soc. B Biol. Sci..

[5] Monlong J., Cossette P., Meloche C., Rouleau G., Girard S.L., Bourque G. (2018). Human copy number variants are enriched in regions of low mappability. Nucleic Acids Res..

[6] Zhan X., Girirajan S., Zhao N., Wu M.C., Ghosh D. (2016). A novel copy number variants kernel association test with application to autism spectrum disorders studies. Bioinformatics.

[7] Brucker A., Lu W., West R.M., Yu Q.Y., Hsiao C.K., Hsiao T.H., Lin C.H., Magnusson P.K., Sullivan P.F., Szatkiewicz J.P. (2020). Association test using Copy Number Profile Curves (CONCUR) enhances power in rare copy number variant analysis. PLoS Comput. Biol..

[8] Esfahani N.M., Catchpoole D., Khan J., Kennedy P.J. (2021). MCKAT, a multi-dimensional copy number variant kernel association test. BMC Bioinform..

[9] Liu D., Ghosh D., Lin X. (2008). Estimation and testing for the effect of a genetic pathway on a disease outcome using logistic kernel machine regression via logistic mixed models. BMC Bioinform..

[10] Wu M.C., Kraft P., Epstein M.P., Taylor D.M., Chanock S.J., Hunter D.J., Lin X. (2010). Powerful SNP-set analysis for case-control genome-wide association studies. Am. J. Hum. Genet..

[11] Davies R.B. (1980). The distribution of a linear combination of *χ*2 random variables. J. R. Stat. Soc. Ser. C Appl. Stat..

[12] Shern J.F., Chen L., Chmielecki J., Wei J.S., Patidar R., Rosenberg M., Ambrogio L., Auclair D., Wang J., Song Y.K. (2014). Comprehensive genomic analysis of Rhabdomyosarcoma reveals a landscape of alterations affecting a common genetic axis in fusion-positive and fusion-negative tumors. Cancer Discov..

[13] Girirajan S., Brkanac Z., Coe B.P., Baker C., Vives L., Vu T.H., Shafer N., Bernier R., Ferrero G.B., Silengo M. (2011). Relative burden of large CNVs on a range of neurodevelopmental phenotypes. PLoS Genet..

[14] El Demellawy D., McGowan-Jordan J., De Nanassy J., Chernetsova E., Nasr A. (2017). Update on molecular findings in rhabdomyosarcoma. Pathology.

[15] Sun X., Guo W., Shen J.K., Mankin H.J., Hornicek F.J., Duan Z. (2015). Rhabdomyosarcoma: Advances in molecular and cellular biology. Sarcoma.

[16] Nishimura R., Takita J., Sato-Otsubo A., Kato M., Koh K., Hanada R., Tanaka Y., Kato K., Maeda D., Fukayama M. (2013). Characterization of genetic lesions in Rhabdomyosarcoma using a high-density single nucleotide polymorphism array. Cancer Sci..

